# Associations of social media and health content use with sexual risk behaviours among adolescents in South Africa

**DOI:** 10.1080/26410397.2023.2267893

**Published:** 2023-11-10

**Authors:** Boladé Hamed Banougnin, Elona Toska, Brendan Maughan-Brown, William Rudgard, Lucas Hertzog, Janina Jochim, Alice Armstrong, Lucie Cluver

**Affiliations:** a Programme Data Analyst, United Nations Population Fund West and Central Africa Regional Office, Dakar, Senegal; Postdoctoral Research Fellow, Centre for Social Science Research, University of Cape Town, South Africa. *Correspondence**:* banougnin@unfpa.org; bolade.banougnin@gmail.com; b Chief Research Officer, Centre for Social Science Research, University of Cape Town, South Africa; Associate Lecturer, Department of Sociology, University of Cape Town, South Africa; [Research Associate] Department of Social Policy and Intervention, University of Oxford, UK; c Chief Research Officer, Southern Africa Labour and Development Research Unit, University of Cape Town, South Africa; d Senior Postdoctoral Research Fellow, Department of Social Policy and Intervention, University of Oxford, UK; e Research Fellow, Centre for Social Science Research, University of Cape Town, South Africa; Research Fellow, Curtin School of Population Health, Faculty of Health Sciences, Curtin University, Perth, Australia; f Postdoctoral Research Fellow, Department of Social Policy and Intervention, University of Oxford, UK; g Regional HIV/AIDS Specialist, UNICEF Eastern and Southern Africa Region, Nairobi, Kenya; h Professor of Child and Family Social Work, Department of Social Policy and Intervention, University of Oxford, UK; Honorary Professor, Department of Psychiatry, University of Cape Town, South Africa

**Keywords:** mobile phone access, social media use, health content use, sexual risk behaviours, HIV

## Abstract

Increasing rates of mobile phone access present potential new opportunities and risks for adolescents’ sexual and reproductive health in resource-poor settings. We investigated associations between mobile phone access/use and sexual risks in a cohort of 10–24-year-olds in South Africa. 1563 adolescents (69% living with HIV) were interviewed in three waves between 2014 and 2018. We assessed mobile phone access and use to search for health content and social media. Self-reported sexual risks included: sex after substance use, unprotected sex, multiple sexual partnerships and inequitable sexual partnerships in the past 12 months. We examined associations between mobile phone access/use and sexual risks using covariate-adjusted mixed-effects logistic regression models. Mobile phone access alone was not associated with any sexual risks. Social media use alone (vs. no mobile phone access) was associated with a significantly increased probability of unprotected sex (adjusted average marginal effects [AMEs] + 4.7 percentage points [ppts], 95% CI 1.6–7.8). However, health content use (vs. no mobile phone access) was associated with significantly decreased probabilities of sex after substance use (AMEs –5.3 ppts, 95% CI –7.4 to –3.2) and unprotected sex (AMEs –7.5 ppts, 95% CI –10.6 to –4.4). Moreover, mobile phone access and health content use were associated with increased risks of multiple sexual partnerships in boys. Health content use was associated with increased risks of inequitable sexual partnerships in adolescents not living with HIV. Results suggest an urgent need for strategies to harness mobile phone use for protection from growing risks due to social media exposure.

## Résumé

Des taux croissants d’accès aux téléphones portables offrent de nouvelles occasions et créent de nouveaux risques potentiels pour la santé sexuelle et reproductive des adolescents dans les environnements à faibles ressources. Nous avons étudié les associations entre l’accès/l’utilisation du téléphone portable et les risques sexuels dans une cohorte de jeunes âgés de 10 à 24 ans en Afrique du Sud. 1563 adolescents (dont 69% vivaient avec le VIH) ont été interrogés en trois vagues entre 2014 et 2018. Nous avons évalué l’accès et l’utilisation du téléphone portable pour rechercher des contenus sur la santé et les médias sociaux. Les risques sexuels autodéclarés comprenaient: les rapports sexuels après consommation de drogues, les rapports sexuels non protégés, les partenariats sexuels multiples et les partenariats sexuels inéquitables au cours des 12 mois précédents. Nous avons examiné les associations entre l’accès/l’utilisation du téléphone portable et les risques sexuels à l’aide de modèles de régression logistique à effets mixtes ajustés en fonction des covariables. L’accès au téléphone portable à lui seul n’était associé à aucun risque sexuel. L’utilisation des médias sociaux seule (par rapport à l’absence d’accès au téléphone portable) était associée à une probabilité significativement accrue de relations sexuelles non protégées (effets marginaux moyens ajustés [AME] + 4,7 points de pourcentage, IC 95% 1,6 à 7,8). Néanmoins, l’utilisation de contenus liés à la santé (par rapport à l’absence d’accès au téléphone portable) était associée à une diminution significative des probabilités d’avoir des rapports sexuels après la consommation de drogues (AME −5,3 points de pourcentage, IC 95% – 7,4 à −3,2) et des relations sexuelles non protégées (AME – 7,5 points de pourcentage, IC 95% – 10,6 à – 4,4). De plus, l’accès à un téléphone portable et l’utilisation de contenus liés à la santé étaient associés à des risques accrus de partenariats sexuels multiples chez les garçons. L’utilisation de contenus liés à la santé était associée à des risques accrus de partenariats sexuels inéquitables chez les adolescents non séropositifs. Les résultats suggèrent un besoin urgent de stratégies visant à exploiter l’utilisation du téléphone portable pour se protéger contre les risques croissants dus à l’exposition aux médias sociaux.

## Resumen

El aumento de las tarifas de acceso a teléfonos móviles presenta posibles oportunidades y riesgos nuevos para la salud sexual y reproductiva de adolescentes en entornos con escasos recursos. Investigamos las asociaciones entre la accesibilidad/uso de teléfonos móviles y los riesgos sexuales en una cohorte de jóvenes entre 10 y 24 años, en Sudáfrica. Entre 2014 y 2018, entrevistamos a 1563 adolescentes (el 69% de quienes vivían con VIH) en tres oleadas. Evaluamos la accesibilidad y el uso de teléfonos móviles para buscar contenido sobre salud y las redes sociales. Algunos de los riesgos sexuales informados por las personas entrevistadas eran: sexo después de la toxicomanía, sexo sin protección, múltiples parejas sexuales y parejas sexuales inequitativas en los últimos 12 meses. Examinamos las asociaciones entre la accesibilidad y el uso de teléfonos móviles y los riesgos sexuales utilizando modelos de regresión logística de efectos mixtos ajustados por covariables. El acceso a teléfonos móviles por sí solo no estaba asociado con riesgos sexuales. El uso de las redes sociales por sí solo (vs. ningún acceso a teléfonos móviles) estaba asociado con un aumento significativo en la probabilidad de tener sexo sin protección (efectos marginales promedio ajustados [AME, por sus siglas en inglés] + 4.7 puntos porcentuales [ppts], IC al 95% de 1.6 a 7.8). Sin embargo, el uso de contenido sobre salud (vs. ningún acceso a teléfonos móviles) estaba asociado con una disminución significativa de las probabilidades de tener sexo después de la toxicomanía (AME –5.3 ppts, IC al 95% de 7.4 a 3.2) y sexo sin protección (AME –7.5 ppts, IC al 95% de 10.6 a 4.4). Además, el acceso a teléfonos móviles y el uso de contenido sobre salud estaban asociados con mayor riesgo de niños varones de tener múltiples parejas sexuales. El uso de contenido sobre salud estaba asociado con mayor riesgo de tener parejas sexuales inequitativas entre adolescentes que no vivían con VIH. Los resultados indican la necesidad urgente de formular estrategias para facilitar el uso de teléfonos móviles para la protección de crecientes riesgos por exposición a las redes sociales.

## Introduction

Sub-Saharan Africa has the largest and fastest-growing youth population, with multiple overlapping sexual and reproductive health (SRH) needs.^1^ In South Africa, the youth population is projected to reach more than 11 million by 2030.^2^ South African youth experience heightened sexual health risk: most new HIV (human immunodeficiency virus) infections occur during adolescence,^3,4^ and one in three girls become pregnant before age 20.^5^

Adolescents and young people have been early and enthusiastic adopters of digital technologies. In 2019, 71% of South African households had a mobile phone user, and 64% had access to the internet,^6^ with youth aged 15–24 years comprising 71% of internet users.^7^ Recent studies have identified the potential of increasing mobile phone access and use to improve adolescents' and young people’s health.^8–11^ Mobile health (m-Health) initiatives may be a key pathway to improve SRH knowledge and HIV prevention among young people in resource-limited settings.^9,12^ Previous studies have shown that m-Health might help prevent adolescents from engaging in risky sexual behaviours as a result of improved SRH knowledge.^9,13^ Findings from a cluster-randomised control trial among 756 females aged 14–24 years in Accra, Ghana, indicated that text messaging improved their SRH knowledge, which in turn led to decreased risks of pregnancy.^14^ Most of m-Health interventions were implemented, taking into account the intersections with adolescents’ sexual and reproductive health and rights in both policy and practice.

However, m-Health interventions remain limited in scope and coverage, without proper evidence of large scaling-up process.^15^ A study on how 4500 young people in Ghana, Malawi and South Africa used mobile phones revealed that the majority of them had never heard of m-Health interventions, let alone participated in them.^16^ Searches via social media and websites have proven to be an innovative way to engage adolescents.^11,17^ Qualitative evidence supports user-driven health content use (rather than campaign-driven health content use) as an effective means of improving health behaviours, ^16–18^ but quantitative research, especially related to sexual risk behaviours, is lacking.

This study attempted to close this gap by focusing on the “informal” uses of m-Health – namely guided by creative and strategic use of mobile phones – for safer sexual risk behaviours.^16^ The paper did not overlook the fact that adolescents are also susceptible to exposure to risks in the online space. A recent meta-analysis showed that frequent use of social media among adolescents is associated with increased risks of drug use, risky sexual practices and violent behaviours.^19^

We aimed to examine the associations of sexual risk behaviours with access to and use of mobile phones among a cohort of adolescents in South Africa, and to assess these associations by sex and HIV status.

## Methods

### Study design

We conducted a prospective cohort study “*Mzantsi Wakho*” amongst 1563 adolescents living with and without HIV in South Africa. This study is reported in accordance with the Strengthening the Reporting of Observational Studies in Epidemiology (STROBE) statement for cohort studies (Appendix 1).^20^

### Study setting and participants

**Figure 1.**
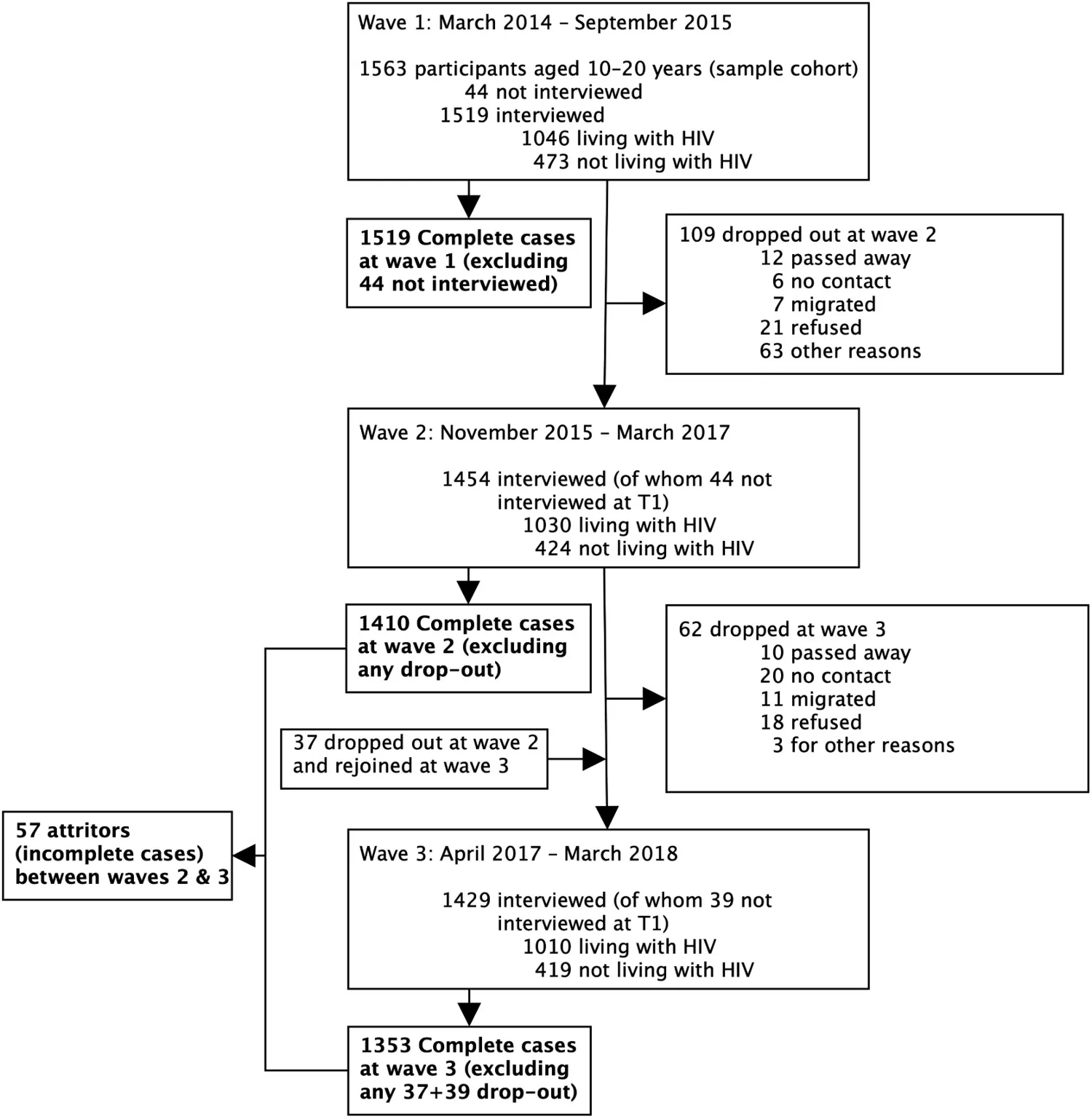
*Mzantsi Wakho* cohort study flow chart. Abbreviations: T1, wave 1; T2, wave 2; T3, wave 3

Research was conducted in 180 communities in a health sub-district in the Eastern Cape Province, South Africa. The Eastern Cape has high levels of poverty and HIV.^21^ The study recruited adolescents living with HIV from 52 primary healthcare clinics in 2014–2015 and 15 additional clinics in 2016–2018, and then recruited neighbouring adolescents not living with HIV in local communities. In the first wave of *Mzantsi Wakho*, which commenced in 2014–2015, 1519 adolescents were successfully interviewed, including 1046 living with HIV and 473 not living with HIV (Figure 1). Wave 2 (1454 interviewed, 1030 of whom were living with HIV; 1410 complete cases) was conducted in 2015–2017 and wave 3 (1429 interviewed, 1010 of whom were living with HIV; 1353 complete cases) in 2017–2018. In waves 2 and 3, participants resided in Eastern Cape, Free State, Gauteng, KwaZulu-Natal, North-West and Western Cape, due to high levels of migration. Ethical protocols were approved by the University of Cape Town (Cape Town, South Africa; CSSR 2013/4, approved 14 April 2013), Oxford University (Oxford, UK; CUREC2/12-21, approved 20 December 2012), Provincial Departments of Health and Education, and all participating healthcare facilities. All adolescents and their primary caregivers provided written informed consent at all survey rounds in Xhosa or English, and POPIA-compliant data management instruments were used across the data lifecycle to protect the personal information of participants.^22^

### Variables/measures

#### Study outcomes

Outcomes were four behaviours important for achieving sexual and reproductive health and preventing HIV among adolescents.^23–26^ All were self-reported for the past 12 months: (1) sex after substance use defined as sexual intercourse when the participant was drunk or used drugs (available in waves 2 and 3); (2) unprotected sex defined as no or infrequent condom use with partners (available in all waves); (3) multiple sexual partnerships defined as two or more sexual partners (available in all waves) and (4) inequitable sexual partnerships defined as sex in exchange for material support of any kind or sexual partner at least 5 years older than participant (available in all waves).

#### Mobile phone access and use

Self-reported data on current access to and use of mobile phone were available in waves 2 and 3. Adolescents were asked if they had a mobile phone (including a smartphone, Apple iPhone, Blackberry, basic phone and SIM card) and if it was their own or shared with someone. In this study, mobile phone access refers to self-reported ownership of a mobile phone with a functional SIM card. Adolescents who owned a mobile phone reported what they used it for and the frequency of use. Mobile phone use options included SMS, WhatsApp, Facebook, Mixit, health information, information about sexual health and HIV-related information. Frequency of use was reported in the questionnaire as “1. Never”, “2. once a month”, “3. once a week”, “4. once a day” and “5. two or more times a day”. Hereafter, we refer to daily or multiple times daily use as (frequent) use of mobile phone (recoded as binary). Mobile phone use measure was then classified as “0. no access”, “1. social media alone” (referring to frequent mobile phone use for SMS, Facebook, Mixit or WhatsApp only) and “2. health content” (referring to frequent mobile phone use for both social media and sexual health or HIV-related information). Access to and use of mobile phone were measured in waves 2 and 3.

#### Covariates

We included eight covariates in our models based on associations identified in existing literature^27^: rural residence; informal housing; household poverty measured as access to the eight highest socially perceived necessities in the nationally representative South African Social Attitudes Survey (enough food, money for school fees, to see a doctor when needed, school uniform, basic clothing, soap, school books and shoes)^28^; being married or in a consensual relationship; age; sex; living with HIV; school enrolment and survey wave. All covariates were categorical variables, except for the participant’s age, which was continuous, and mean-centred before analysis to facilitate interpretations. Covariates changed for very few individuals in the data. Therefore, all covariates, except for the survey wave, were measured at baseline (between March 2014 and September 2015).

### Data and bias

Participants who dropped out of the study between waves 2 and 3 (*n* = 57) did not differ systematically from participants who completed both waves (*n* = 1353) (Appendix 2) suggesting no strong evidence of attrition bias.^29–31^ Little’s χ^2^ test, with the presence of covariates and unequal variances between different missing-value patterns, indicated that data were not missing completely at random (Appendix 3).^32^ Frequencies showed that more missing data occurred in wave 2 than in wave 3. The proportion of missing data on sex after substance use was 13.5% in wave 2 and 6.5% in wave 3; the proportion of missing data on inequitable sexual partnerships ranged from 3.8% in wave 2 to 6.5% in wave 3. There was no missing data on mobile phone access and use (neither in wave 2 nor in wave 3). The amount of missing data for covariates at baseline was 0.1% for household poverty and informal housing. There was no missing data on sex, age, rural residence, relationship status, school enrollment and HIV status. To maximise statistical power while minimising bias, we imputed missing data ten times in wide format using chained equations method.^33–35^ Imputation models included all aforementioned covariates and auxiliary variables that were predictive of sexual risk behaviours.

### Statistical methods

We first described sociodemographic characteristics, mobile phone access and use, individual sexual risk behaviours in the full sample, then by adolescent sex and HIV status. The correlations between individual outcomes were weak or moderate. We reported Spearman’s bivariate correlation coefficients between outcome variables in Appendix 4. We reported the descriptive statistics for the original sample (i.e. data with missing values). Second, we used mixed effects logistic regression models on imputed data to account for repeated measures.^36^ A summary of the models is reported in Appendix 5. We estimated the overall association of individual sexual risk behaviours with mobile phone access (Model 1) and mobile phone use for social media alone and health content (Model 2), adjusting for baseline covariates. We reported average marginal effects from the models to estimate associations of individual sexual risk behaviours with mobile access and use. We also estimated the separate associations of individual sexual risk behaviours with mobile phone access (Appendix 5, Model 3) and mobile phone use (Appendix 5, Model 4) for two sub-groups: boys and girls, and for adolescents living with and without HIV. To prevent underpowered (stratified) analyses, we estimated average marginal effects (AMEs) in all four combinations (of boys or girls, living with HIV or not) using three-way interaction terms (between participant’s sex, HIV status and mobile phone access/use). Third, we conducted sensitivity analyses, comparing the results in an analysis that included only complete cases (from data with no imputations) to results from imputed data. All analyses were done using Stata 17 ^37^. Statistical significance was defined a priori as *p* < 0.05.

## Results

Table 1 shows baseline socio-demographic characteristics of participants (*n* = 1410), access to and use of mobile phones (in waves 2 and 3), and sexual risk behaviours (in waves 2 and 3). 69% of participants recruited at baseline were living with HIV. The baseline mean age of respondents was 13.8 years. 57% were female, 27% resided in a rural area, 18% were living in informal houses and 67% were in poor households. Nearly one-third of the sample was in a relationship and 94% were enrolled in school. In wave 2, about half of participants (50%) had access to a mobile phone (including 43% for boys, 56% for girls, 57% for adolescents not living with HIV and 48% for adolescents living with HIV), 44% used mobile phones for social media alone (including 24% for boys, 41% for girls, 49% for adolescents not living with HIV and 36% for adolescents living with HIV) and only 17% for health content (including 20% for boys, 15% for girls, 8% for adolescents not living with HIV and 21% for adolescents living with HIV). In wave 3, there was a slight increase in the proportion of mobile phone access (53%) including 47% for boys, 65% for girls, 63% for adolescents not living with HIV and 55% for adolescents living with HIV; 34% used mobile phone for social media alone (including 25% for boys, 43% for girls, 55% for adolescents not living with HIV and 45% for adolescents living with HIV) and 23% used mobile phone for health content (including 22% for boys, 24% for girls, 7% for adolescents not living with HIV and 30% for adolescents living with HIV).

**Table 1.** Descriptive characteristics of the study population

	Sample size	All participants	Boys	Girls	Adolescents not living with HIV	Adolescents living with HIV
**Wave 1, 2014–2015**						
Female	1410	56.7%	..	..	60.3%	55.1%
ALHIV	1410	69.4%	72.0%	67.5%	..	..
Age	1410	13.8 (0.08)	13.2 (0.11)	14.3 (0.11)	14.3 (0.10)	13.6 (0.6)
Rural residence	1410	27.4%	25.5%	28.9%	29.0%	26.8%
Informal housing	1409	18.0%	15.7%	19.8%	16.5%	18.7%
Poverty	1409	67.2%	64.0%	69.7%	65.6%	67.9%
In a relationship	1410	29.3%	25.4%	32.3%	40.6%	24.3%
School enrolment	1410	94.3%	98.4%	91.2%	94.7%	94.2%
**Wave 2, 2015–2017**						
Use	1410					
No access		49.6%	56.6%	44.2%	42.9%	52.5%
Social media alone		33.3%	23.6%	40.7%	49.2%	36.3%
Health content		17.1%	19.8%	15.1%	7.9%	21.2%
Sex after substance use	1221	7.2%	8.4%	6.3%	11.5%	5.3%
Unprotected sex	1410	14.7%	9.0%	19.0%	20.6%	12.1%
Multiple sexual partnerships	1410	15.1%	16.7%	13.9%	18.3%	13.7%
Inequitable sexual partnership	1352	10.7%	9.7%	11.4%	10.4%	10.8%
**Wave 3, 2017–2018**						
Use	1353					
No access		42.7%	53.2%	34.7%	37.4%	45.1%
Social media alone		34.1%	24.7%	41.3%	55.2%	44.5%
Health content		23.2%	22.1%	24.0%	7.4%	30.3%
Sex after substance use	1265	7.5%	9.3%	6.2%	11.1%	5.9%
Unprotected sex	1353	21.7%	12.3%	28.9%	31.9%	17.0%
Multiple sexual partnerships	1353	16.0%	19.6%	13.3%	20.2%	14.1%
Inequitable sexual partnership	1237	9.9%	6.2%	12.7%	12.9%	8.6%

Note. Data are % or mean (SD) unless otherwise specified. Abbreviations: SD, standard deviation.

**Figure 2.**
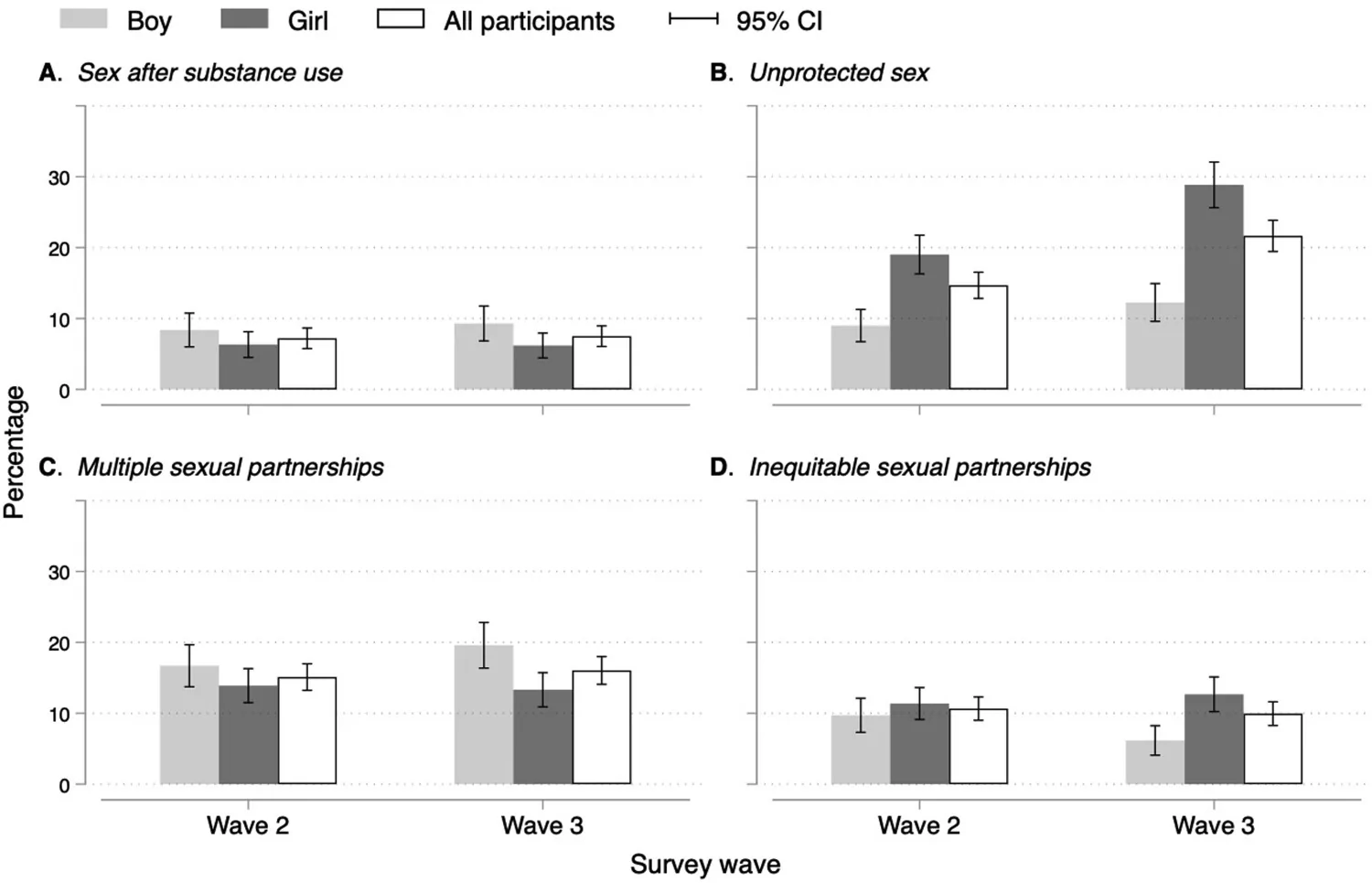
Sexual risk behaviours by participant's sex across waves. Abbreviations: Cl, confidence interval

**Figure 3.**
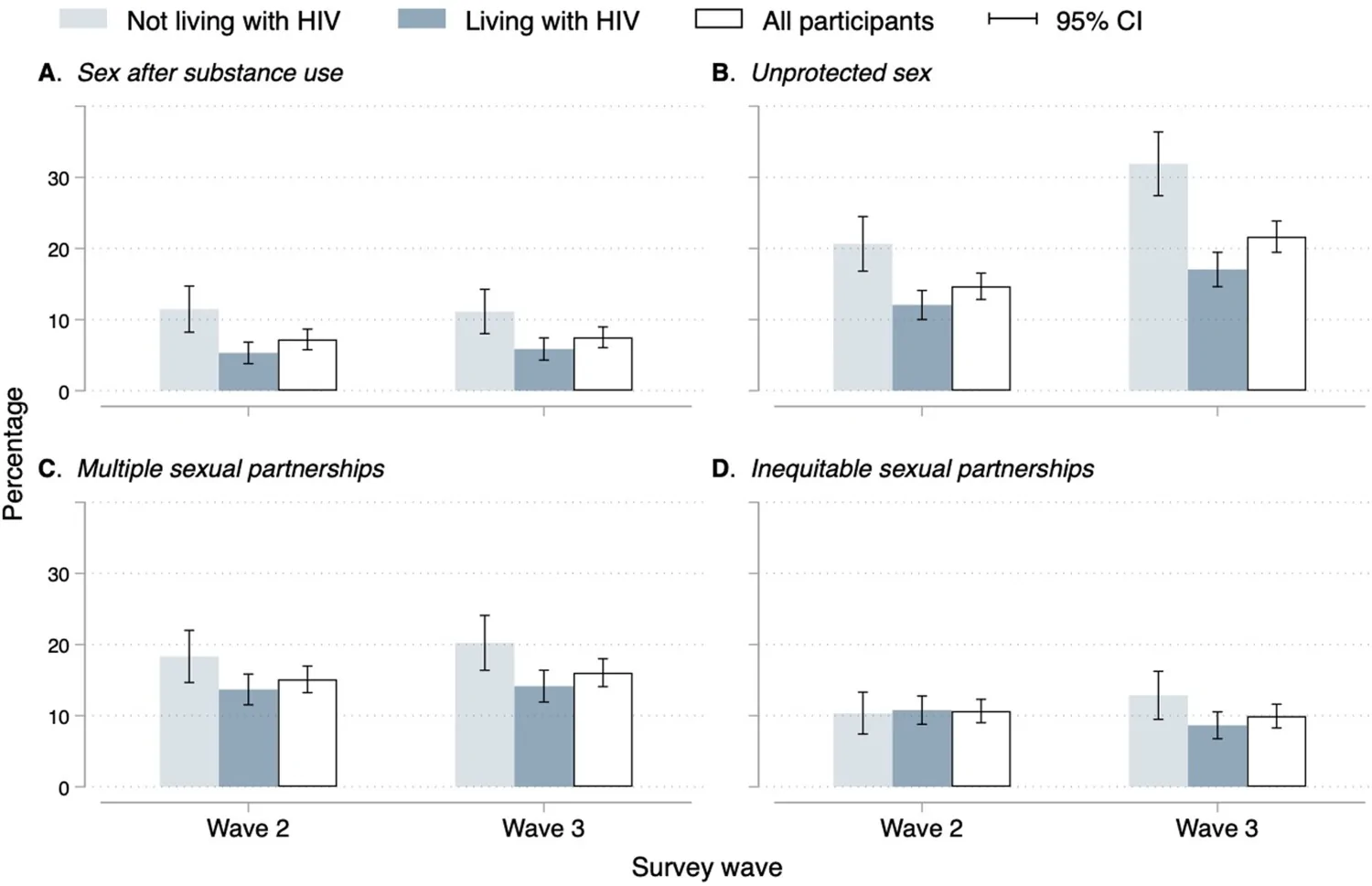
Sexual risk behaviours by participant's HIV status across waves. Abbreviations: Cl, confidence interval. HIV, human immunodeficiency virus . HIV-, adolescents not living with HIV. HIV+, adolescents living with HIV

Sexual risk behaviours were similar across waves (Figure 2), except for unprotected sex which increased from 15% in wave 2 to 22% in wave 3 (Figure 2B). For boys, there was no change in the prevalence of any of the sexual risk behaviours between waves 2 and 3. For girls, the prevalence of unprotected sex increased from 19% in wave 2 to 29% in wave 3 (Figure 2B). The proportion of self-reported unprotected sex and sex after substance use was significantly higher among adolescents not living with HIV than among adolescents living with HIV in waves 2 and 3 (Figure 3A and Figure 3B). There was a significant increase in the proportion of self-reported unprotected sex between waves 2 and 3 among adolescents not living with HIV (from 21% in wave 2 to 32% in wave 3) and adolescents living with HIV (from 12% in wave 2 to 17% in wave 3) (Figure 3B). In wave 3, the proportion of self-reported multiple sexual partnerships was higher among adolescents not living with HIV (20%) than among adolescents living HIV (14%) (Figure 3C).

We fit two multivariable models to determine the association of individual sexual risk behaviours with mobile phone access (Table 2, Model 1) and mobile phone use (Table 2, Model 2). We found no evidence of an association between mobile phone access and sexual risk behaviours after adjusting for covariates.

**Table 2.** Multivariable association between mobile phone access and use and self-reported sexual risk behaviours. Results from mixed-effects logistic regression models on ten imputed datasets (*n* = 1353)

	Sex after substance use		Unprotected sex		Multiple sexual partnerships		Inequitable sexual partnerships	
	Adjusted average marginal effects (95% CI)	*p*-value	Adjusted average marginal effects (95% CI)	*p*-value	Adjusted average marginal effects (95% CI)	*p*-value	Adjusted average marginal effects (95% CI)	*p*-value
**Model 1 (Mobile phone access vs. no access)**								
No mobile phone access (Reference)	0		0		0		0	
Mobile phone access	–0.0056 (–0.0269 to 0.0157)	0.605	0.0081 (–0.0187 to 0.0350)	0.552	0.0146 (–0.0110 to 0.0404)	0.264	0.0003 (–0.0245 to 0.0251)	0.981
**Model 2 (Social media alone and health content vs. no mobile phone access)**								
No mobile phone access (Reference)	0		0		0		0	
Social media alone	0.0235 (–0.0016 to 0.0487)	0.066	**0.0468** **(0.0156** **to 0.0780)**	**0.003**	0.0177 (–0.0108 to 0.0461)	0.223	0.0015 (–0.0258 to 0.0287)	0.916
Health content use	**–0.0532** **(–0.0739** **to –0.0324)**	**<0**.**001**	**–0.0750** **(–0.1063** **to –0.0436)**	**<0.001**	0.0095 (–0.0230 to 0.0420)	0.569	–0.0016 (–0.0323 to 0.0290)	0.917

Data are average marginal effects (and 95% CI) adjusted for participant’s sex, rural residence, informal housing, HIV status, household poverty and marital status. Data were obtained from mixed-effects logistic regression models on ten imputed data (*n* = 1353). Model 1 presents data from regression of individual sexual risk behaviours on mobile phone access (access vs. no access), adjusting for covariates. Model 2 presents data from regression of individual sexual risk behaviours on mobile phone use (social media alone and health content vs. no access), adjusting for covariates. Abbreviations: CI, confidence interval.

Social media use alone (as compared to no mobile phone access) was associated with a significantly increased probability of unprotected sex (AMEs +4.7 percentage points [ppts], 95% CI 1.6 to 7.8, *p *= 0.003). Health content use (as compared to no mobile phone access) was associated with a significantly decreased probability of sex after substance use (AMEs –5.3 ppts, 95% CI –7.4 to –3.2, *p *< 0.001) and decreased probability of unprotected sex (AMEs –7.5 ppts, 95% CI –10.6 to –4.4, *p *< 0.001).

Then we estimated separate effects of mobile phone access (Table 3, Model 3) and mobile phone use (Table 3, Model 4) for boys, girls and adolescents living with and without HIV. Mobile phone access was associated with a significantly increased probability of self-reported multiple sexual partnerships among boys only (AMEs 5.5 ppts, 95% CI 1.1–9.6, *p *= 0.013). In all four categories, adolescents who used mobile phones for health content had significantly lower probabilities of reporting unprotected sex (for boys: AMEs –6.8 ppts, 95% CI –11.4 to –2.2, *p *= 0.004; for girls: AMEs –7.9 ppts, 95% CI –12.5 to –3.2, *p *= 0.001; for adolescents not living with HIV: AMEs –10.0 ppts, 95% CI –17.0 to –3.1, *p *= 0.005; for adolescents living with HIV: AMEs –6.0 ppts, 95% CI –9.4 to –2.6, *p *= 0.001) than adolescents with no access to a mobile phone. In all four categories except for adolescents not living with HIV, health content use (*vs*. no mobile phone access) was associated with significantly decreased probabilities of reporting sex after substance use (for boys: AMEs –6.8 ppts, 95% CI –11.5 to –2.2, *p *= 0.004; for girls: AMEs –3.4 ppts, 95% CI –6.2 to –0.6, *p *= 0.018; for adolescents living with HIV: AMEs –6.4 ppts, 95% CI –8.7 to –4.1, *p *< 0.001). Health content use (*vs*. no mobile phone access) was associated with a significantly increased probability of reporting multiple sexual partnerships for boys only (AMEs 7.6 ppts, 95% CI 1.5–13.7, *p *= 0.015). Health content use (*vs* no mobile phone access) was associated with a significantly increased probability of reporting inequitable sexual partnerships for adolescents not living with HIV only (AMEs 8.7 ppts, 95% CI 0.9–16.6, *p *= 0.029). Social media use of mobile phone (*vs*. no mobile phone access) was associated with significantly increased probabilities of sex after substance use among boys (AMEs 6.6 ppts, 95% CI 1.2–12.0, *p *= 0.017) and adolescents living with HIV (AMEs 4.3 ppts, 95% CI 0.7–7.8, *p *= 0.018). Social media use of mobile phone (*vs*. no mobile phone access) was associated with significantly increased probabilities of self-reported unprotected sex for girls (AMEs 6.2 ppts, 95% CI 1.8–10.5, *p *= 0.005) and adolescents living with HIV (AMEs 9.4 ppts, 95% CI 5.3–13.4, *p *< 0.001).

**Table 3.** Comparisons of the multivariable association between mobile phone access and use and self-reported sexual risk behaviours for boys, girls, adolescents living with and without HIV. Results from mixed-effects logistic regression models on ten imputed datasets (*n* = 1353 including 587 boys, 766 girls, 933 living with HIV, and 420 not living with HIV)

	Sex after substance use		Unprotected sex		Multiple sexual partnerships		Inequitable sexual partnerships	
	Adjusted average marginal effects (95% CI) *p*-value		Adjusted average marginal effects (95% CI)	*p*-value	Adjusted average marginal effects (95% CI)	*p*-value	Adjusted average marginal effects (95% CI)	*p*-value
**Model 3 (Mobile phone access vs. no access)**								
*Among boys*								
No mobile phone access (Reference)	0		0		0		0	
Mobile phone access	–0.0060 (–0.0447 to 0.0327)	0.760	–0.0019 (–0.0396 to 0.0359)	0.923	**0.0535** **(0.0111** **to 0.0959)**	**0**.**013**	–0.0068 (–0.0462 to 0.0326)	0.735
*Among girls*								
No mobile phone access (Reference)	0		0		0		0	
Mobile phone access	–0.0055 (–0.0300 to 0.0190)	0.660	0.0135 (–0.0237 to 0.0508)	0.477	–0.0144 (–0.0454 to 0.0166)	0.362	0.0053 (–0.0257 to 0.0363)	0.738
*Among adolescents not living with HIV*								
No mobile phone access (Reference)	0		0		0		0	
Mobile phone access	0.0021 (–0.0372 to 0.0413)	0.917	–0.0313 (–0.0810 to 0.0185)	0.218	0.0226 (–0.0227 to 0.0679)	0.328	0.0008 (–0.0401 to 0.0418)	0.968
*Among adolescents living with HIV*								
No mobile phone access (Reference)	0		0		0		0	
Mobile phone access	–0.0102 (–0.0355 to 0.0151)	0.428	0.0292 (–0.0024 to 0.0608)	0.071	0.0059 (–0.0262 to 0.0380)	0.719	0.0015 (–0.0292 to 0.0322)	0.923
**Model 4 (Social media use and health content use vs. no mobile phone access)**								
*Among boys*								
No mobile phone access (Reference)	0		0		0		0	
Social media use	**0.0659** **(0.0117** **to 0.1201)**	**0**.**017**	0.0431 (–0.0049 to 0.0912)	0.078	0.0441 (–0.0057 to 0.0940)	0.082	0.0035 (–0.0450 to 0.0520)	0.888
Health content use	**–0.0683** **(–0.1150** **to –0.0216)**	**0**.**004**	**–0.0683** **(–0.1143** **to –0.0222)**	**0**.**004**	**0.0759** **(0.0145** **to 0.1373)**	**0**.**015**	0.0106 (–0.0492 to 0.0703)	0.729
*Among girls*								
No mobile phone access (Reference)	0		0		0		0	
Social media use	0.0069 (–0.0197 to 0.0334)	0.611	**0.0617** **(0.0182** **to 0.1051)**	**0**.**005**	–0.0061 (–0.0390 to 0.0268)	0.716	0.0096 (–0.0239 to 0.0430)	0.575
Health content use	**–0.0340** **(–0.0621** **to –0.0059)**	**0**.**018**	**–0.0786** **(–0.1250** **to –0.0322)**	**0**.**001**	–0.0284 (–0.0689 to 0.0121)	0.169	0.0243 (–0.0199 to 0.0684)	0.281
*Among adolescents not living with HIV*								
No mobile phone access (Reference)	0		0		0		0	
Social media use	0.0059 (–0.0349 to 0.0467)	0.775	–0.0208 (–0.0729 to 0.0314)	0.435	0.0179 (–0.0283 to 0.0642)	0.447	–0.0132 (–0.0543 to 0.0280)	0.531
Health content use	–0.0180 (–0.0714 to 0.0355)	0.510	**–0.1002** **(–0.1699** **to –0.0305)**	**0**.**005**	0.0503 (–0.0288 to 0.1295)	0.213	**0.0874** **(0.0087** **to 0.1662)**	**0**.**029**
*Among adolescents living with HIV*								
No mobile phone access (Reference)	0		0		0		0	
Social media use	**0.0429** **(0.0074** **to 0.0784)**	**0**.**018**	**0.0939** **(0.0533** **to 0.1344)**	**<0**.**001**	0.0114 (–0.0257 to 0.0486)	0.546	0.0200 (–0.0168 to 0.0568)	0.286
Health content use	**–0.0641** **(–0.0873** **to –0.0408)**	**<0**.**001**	**–0.0600** **(–0.0944** **to –0.0256)**	**0.001**	–0.0100 (–0.0476 to 0.0277)	0.606	–0.0202 (–0.0551 to 0.0146)	0.256

Data are average marginal effects (and 95% CI) from models with interaction between participant’s sex, HIV status and mobile phone access (Model 3) or mobile phone use (Model 4), adjusting for rural residence, informal housing, household poverty and marital status. Data were obtained from mixed-effects logistic regression models on ten imputed data (*n* = 1353 including 587 boys, 766 girls, 933 living with HIV and 420 not living with HIV).

We conducted a sensitivity analysis with complete cases and compared to the imputed dataset. The results from the sensitivity analysis and main analysis were similar, confirming overall results (Appendix 6a and 6b).

## Discussion

This study provides valuable insight into the rates of mobile phone use alongside sexual risk behaviours of young people who are taking the initiative to “use m-Health” informally, in a high-poverty context in South Africa. We found that 57% of adolescents in our study had access to a mobile phone in 2018; this is consistent with the national average of 55% of mobile phone access in 2019.^7^ Social media use alone was almost ubiquitous, but less than a quarter of adolescent mobile phone owners had accessed health content in the past 12 months.

Our findings showed no association between mobile phone access and self-reported sexual risk behaviours. However, social media use alone was associated with an increased probability of unprotected sex and sex after substance use among adolescents living with HIV – with consequent risks of HIV exposure for their sexual partners. In contrast, accessing digital content on health or HIV (informal m-Health) – even alongside social media use only – was protective against sexual risk and was associated with lower rates of sex after substance use and unprotected sex.

These findings support and advance the existing literature. Two recent reviews found no associations between access to a mobile phone and adolescent sexual risk, suggesting that access alone to mobile phones is insufficient to improve adolescents’ health outcomes.^11,38^ Our study supports these conclusions, and additionally examines the relationship amongst a large group of adolescents living with HIV, finding that mobile phone access in itself neither reduces nor increases sexual risks for this group.

Our study found that exclusive use of social media (without concurrent use of health content) is associated with increased unprotected sex. This quantitative evidence adds to qualitative studies and reviews from high-income settings finding that social media use can negatively impact adolescent self-esteem and contribute to high-risk behaviours.^39–42^ In this South African sample, the increase in unprotected sex amongst adolescents living with HIV and using mobile phones for social media alone suggests that this group may be particularly vulnerable to damaging effects of social media (alone and without access to any health-related information).

Two meta-analyses and a recent systematic review of mobile health interventions report overall benefits for adolescent health behaviours, including sexual health.^13,43,44^ This study adds to the evidence base by demonstrating beneficial associations of real-world use of health content, in almost all cases alongside social media, amongst adolescents living with and without HIV in South Africa. However, across both waves and in all subgroups, less than a quarter of adolescents had accessed any health content at all on their mobile phones in the past year. This suggests that while health content may be valuable, its uptake amongst this very high-risk group remains low, with consequent need for large-scale interventions that increase access and use of age-appropriate up-to-date sexual and reproductive health intervention. This also includes access to health content via social media.

We note several limitations. First, the use of retrospective self-report measures of sexual risk behaviours may increase bias related to social desirability and recall biases. However, self-report is currently the only feasible way to measure most adolescent sexual risk behaviours. To mitigate measurement errors the study used widely validated measures in previous adolescent sexual health research in South Africa. Second, despite the longitudinal design, causality between the access to and use of mobile phones and sexual risk behaviours cannot be confirmed. Third, although we found no systematic differences between participants who completed both survey rounds and those who dropped out, we cannot fully rule out possible biases from unmeasured sources of confounding and attrition. Other caveats include potential bias due to data not completely missing at random. However, we used multiple imputations to account for biases in missing data. We also repeated the analysis in a sensitivity analysis using complete cases analysis and again found similar results. Fifth, we fit multivariable mixed-effects logistic regression for each outcome separately, therefore, assuming that the covariances among random effects across all sexual risk behaviours outcomes and the covariances among the residuals equal zero. Nevertheless, our findings from sensitivity analyses do not deviate from estimates from multivariable models.

The study also has a number of strengths. It adds to evidence from formal m-Health interventions, by examining a real-life sample of adolescents who use (and do not use) mobile phones informally to improve their SRHR knowledge and how they engage in safer sexual practices. In the absence of large-scale formal m-Health programmes in resource-limited settings, data from informal m-Health can inform development of adolescents’ SRHR and HIV prevention programmes. However, the measure of informal m-Health used in this study is insufficiently detailed to assess the complexity of its associations with adolescents’ sexual risk behaviours. Future surveys should attempt to collect data on mobile phone use in relation to sexual risk behaviours. We collected longitudinal data from adolescents living with and without HIV and analysed a sample of adolescent girls and boys, living with HIV or not, adding to our understanding of informal use of social media and health content amongst and comparing between these important groups.

This study suggests that mobile phones can be a medium of both risk and resilience for adolescents in Southern Africa. Social media use only – without concurrent health content use – was associated with increased sexual risk. Health content use was protective, even in the context of concurrent social media use, but under-utilised. This highlights important next steps for programming: to identify approaches that increase informal m-Health use amongst adolescents and to deliver and assess these in real-world settings. For example, UNICEF and partners have recently proposed a set of toolkits to address sexual and reproductive health and HIV prevention needs.^45^ As mobile and internet access increases exponentially in Africa over the next decade, it is essential that we minimise associated risks, and capitalise on the potential of mobile phones to improve adolescent sexual and reproductive health.

## Supplementary Material

Supplemental Material: Appendices 1-6

## Data Availability

Prospective users, policymakers/government agencies/researchers (internal/external) will be required to contact the study team to discuss and plan the use of data. Research data will be available on request subject to participant consent and having completed all necessary documentation. All data requests should be sent to the Elona Toska (elona.toska@uct.ac.za) or William Rudgard (william.rudgard@spi.ox.uk).

## Appendices

**Appendix 1:** STROBE Statement—Checklist of items that should be included in reports of ***cohort studies***

	Item No	Recommendation	Page No
**Title and abstract**	1	(*a*) Indicate the study’s design with a commonly used term in the title or the abstract	1
		(*b*) Provide in the abstract an informative and balanced summary of what was done and what was found	2
Introduction			
Background/rationale	2	Explain the scientific background and rationale for the investigation being reported	3
Objectives	3	State specific objectives, including any prespecified hypotheses	3
Methods			
Study design	4	Present key elements of study design early in the paper	4
Setting	5	Describe the setting, locations, and relevant dates, including periods of recruitment, exposure, follow-up, and data collection	4/5
Participants	6	(*a*) Give the eligibility criteria, and the sources and methods of selection of participants. Describe methods of follow-up	4/5
		(*b*) For matched studies, give matching criteria and number of exposed and unexposed	N/A
Variables	7	Clearly define all outcomes, exposures, predictors, potential confounders, and effect modifiers. Give diagnostic criteria, if applicable	4/6
Data sources/ measurement	8*	* *For each variable of interest, give sources of data and details of methods of assessment (measurement). Describe comparability of assessment methods if there is more than one group	4/6
Bias	9	Describe any efforts to address potential sources of bias	6
Study size	10	Explain how the study size was arrived at	5
Quantitative variables	11	Explain how quantitative variables were handled in the analyses. If applicable, describe which groupings were chosen and why	6/7
Statistical methods	12	(*a*) Describe all statistical methods, including those used to control for confounding	6/7
		(*b*) Describe any methods used to examine subgroups and interactions	6/7
		(*c*) Explain how missing data were addressed	6
		(*d*) If applicable, explain how loss to follow-up was addressed	6
		(*e*) Describe any sensitivity analyses	6/7
Results			
Participants	13*	(a) Report numbers of individuals at each stage of study—eg numbers potentially eligible, examined for eligibility, confirmed eligible, included in the study, completing follow-up, and analysed	7/8
		(b) Give reasons for non-participation at each stage	5/6
		(c) Consider use of a flow diagram	5
Descriptive data	14*	(a) Give characteristics of study participants (eg demographic, clinical, social) and information on exposures and potential confounders	7/8
		(b) Indicate number of participants with missing data for each variable of interest	N/A
		(c) Summarise follow-up time (e.g., average and total amount)	N/A
Outcome data	15*	Report numbers of outcome events or summary measures over time	8/9
Main results	16	(*a*) Give unadjusted estimates and, if applicable, confounder-adjusted estimates and their precision (eg, 95% confidence interval). Make clear which confounders were adjusted for and why they were included	10/11
		(*b*) Report category boundaries when continuous variables were categorized	10/11
		(*c*) If relevant, consider translating estimates of relative risk into absolute risk for a meaningful time period	N/A
Other analyses	17	Report other analyses done—e.g., analyses of subgroups and interactions, and sensitivity analyses	10/13
**Discussion**			
Key results	18	Summarise key results with reference to study objectives	13/15
Limitations	19	Discuss limitations of the study, taking into account sources of potential bias or imprecision. Discuss both direction and magnitude of any potential bias	13/15
Interpretation	20	Give a cautious overall interpretation of results considering objectives, limitations, multiplicity of analyses, results from similar studies, and other relevant evidence	13/15
Generalisability	21	Discuss the generalisability (external validity) of the study results	14/15
**Other information**			
Funding	22	Give the source of funding and the role of the funders for the present study and, if applicable, for the original study on which the present article is based	19

*Give information separately for exposed and unexposed groups.

**Note:** An Explanation and Elaboration article discusses each checklist item and gives methodological background and published examples of transparent reporting. The STROBE checklist is best used in conjunction with this article (freely available on the Web sites of PLoS Medicine at http://www.plosmedicine.org/, Annals of Internal Medicine at http://www.annals.org/, and Epidemiology at http://www.epidem.com/). Information on the STROBE Initiative is available at http://www.strobe-statement.org.

**Appendix 2:** Comparison between participants interviewed in both waves (2 and 3) and those who dropped out of the study (between wave 2 and 3)

	Total (N=1,410)	Included (N=1,353)	Excluded (N=57)	p-value
Rural residence	27.4%	27.5%	26.3%	0.85
Informal housing	18.0%	17.8%	22.8%	0.34
Poverty	32.8%	32.9%	29.8%	0.63
Age (in years)	13.8 (2.9)	13.8 (3.0)	14.5 (2.7)	0.062
Sex				0.85
Male	43.3%	43.4%	42.1%	
Female	56.7%	56.6%	57.9%	
ALHIV	69.4%	69.0%	80.7%	0.059
In a relationship	29.3%	29.3%	29.8%	0.93
School enrolment	94.3%	94.4%	93.0%	0.65
Mobile phone use				0.015
No access	49.6%	49.8%	43.9%	
Social media only	33.3%	32.6%	49.1%	
Health content use	17.1%	17.6%	7.0%	
Sex after substance use	7.2%	7.1%	9.8%	0.46
Multiple sexual partnerships	15.1%	15.2%	12.3%	0.54
Unprotected sex	14.7%	14.5%	19.3%	0.31
Inequitable sexual partnerships	10.7%	10.8%	5.9%	0.26

**Appendix 3:** Missing data imputations

	Missing – N (%)
**Wave 1**	
Female	0 (0%)
ALHIV	0 (0%)
Age	0 (0%)
Rural residence	0 (0%)
Informal housing	1 (0.07%)
Poverty	1 (0.07%)
In a relationship	0 (0%)
**Wave 2, 2015–2017**	
Mobile phone use	0 (0%)
Sex after substance use	183 (13.53%)
Unprotected sex	0 (0%)
Multiple sexual partnerships	0 (0%)
Inequitable sexual partnership (defined as self-report of sexual intercourse with partners 5+ years older than participants or in exchange of material gifts.)	52 (3.84%)
**Wave 3, 2017–2018**	
Mobile phone use	0 (0%)
Sex after substance use	88 (6.50%)
Unprotected sex	0 (0%)
Multiple sexual partnerships	0 (0%)
Inequitable sexual partnership	116 (8.57%)

Determining whether the data on outcomes are MCAR

Little's χ² test indicated that data were not missing completely at random Chi-square = 33.97 (with degrees of freedom 11) in wave 2 and 58.02 in wave 3 (with degrees of freedom 11)

**Appendix 4:** Spearman’s bivariate correlation coefficients (95% CI; p-value) between outcome variables. Below the diagonal (value 1) are between-participants correlations and above the diagonal are within-participants correlations

	Sex after substance use	Unprotected sex	Multiple sexual partnerships	Inequitable sexual partnerships
Sex after substance use	1	0.06 (0.02 to 0.10); p=0.003	0.16 (0.12 to 0.20); p<0.001	0.04 (0.00 to 0.08); p=0.043
Unprotected sex	0.25 (0.20 to 0.30); p<0.001	1	0.02 (-0.02 to 0.06); p=0.288	0.01 (-0.03 to 0.05); p=0.680
Multiple sexual partnerships	0.44 (0.40 to 0.48); p<0.001	0.38 (0.34 to 0.43); p<0.001	1	0.17 (0.13 to 0.21); p<0.001
Inequitable sexual partnerships	0.27 (0.22 to 0.32); p<0.001	0.39 (0.34 to 0.43); p<0.001	0.47 (0.42 to 0.51); p<0.001	1

**Appendix 5:** Summary of statistical models

Model	Main association	Adjustment variables
Model 1	Average marginal effects from the multivariable mixed-effects logistic regression of individual sexual risk behaviours outcomes on mobile phone access (access vs. no access).	Participant’s sex, mean-centred age, HIV status, relationship status, and household poverty, rural residence, informal housing, school enrolment, and survey wave.
Model 2	Average marginal effects from the multivariable mixed-effects logistic regression of individual sexual risk behaviours outcomes on mobile phone use (social media use alone and health content use vs. no access).	Participant’s sex, mean-centred age, HIV status, relationship status, and household poverty, rural residence, informal housing, school enrolment, and survey wave.
Model 3	Average marginal effects from the multivariable mixed-effects logistic regression of individual sexual risk behaviours outcomes on mobile phone access (access vs. no access), participant’s sex, interaction between ownership, participant’s sex, and HIV status. Model 3 presents the average marginal effects from the association between mobile phone access and individual sexual risk behaviours outcomes for boys, girls, adolescents living with HIV, and not living with HIV.	Participant’s mean-centred age, relationship status, and household poverty, rural residence, informal housing, school enrolment, and survey wave.
Model 4	Average marginal effects from the multivariable mixed-effects logistic regression of individual sexual risk behaviours outcomes on mobile phone use (social media use alone and health content use vs. no access), participant’s sex, interaction between ownership, participant’s sex, and HIV status. Model 4 presents the average marginal effects from the association between mobile phone use and individual sexual risk behaviours outcomes for boys, girls, adolescents living with HIV, and not living with HIV.	Participant’s mean-centred age, relationship status, and household poverty, rural residence, informal housing, school enrolment, and survey wave.

**Appendix 6a:** **Sensitivity analyses**. Multivariable association between mobile phone access and use and self-reported sexual risk behaviours. Results from mixed-effects logistic regression models on complete cases

	Sex after substance use		Unprotected sex		Multiple sexual partnerships		Inequitable sexual partnerships	
	Adjusted average marginal effects (95% CI)	p–value	Adjusted average marginal effects (95% CI)	p–value	Adjusted average marginal effects (95% CI)	p–value	Adjusted average marginal effects (95% CI)	p–value
**Model 1 (Mobile phone access vs. no access)**								
No mobile phone access (Reference)	0	.	0	.	0	.	0	.
Mobile phone access	–0.0065 (–0.0271 to 0.0140)	0.551	0.0062 (–0.0201 to 0.0335)	0.611	0.0153 (–0.0109 to 0.0411)	0.242	0.0005 (–0.0247 to 0.0250)	0.961
**Model 2 (Social media alone and health content vs. no mobile phone access)**								
No mobile phone access (Reference)	0	.	0	.	0	.	0	.
Social media alone	0.0252 (–0.0021 to 0.0482)	0.077	0.0456 (0.0142 to 0.0767)	0.005	0.0184 (–0.0105 to 0.0465)	0.209	0.0017 (–0.0251 to 0.0285)	0.905
Health content use	–0.0538 (–0.0746 to –0.0331)	<0.001	–0.0758 (–0.1072 to –0.0445)	<0.001	0.0107 (–0.0220 to 0.0433)	0.534	–0.0016 (–0.0322 to 0.0291)	0.919
N	1332		1351		1351		1333	

**Appendix 6b:** Sensitivity analyses. Comparisons of the multivariable association between mobile phone access and use and self-reported sexual risk behaviours for boys, girls, adolescents living with and without HIV. Results from mixed-effects logistic regression models on complete cases

	Sex after substance use		Unprotected sex		Multiple sexualpartnerships		Inequitable sexualpartnerships	
	Adjustedaveragemarginaleffects(95% CI)	p–value	Adjustedaveragemarginaleffects(95% CI)	p–value	Adjustedaveragemarginaleffects(95% CI)	p–value	Adjustedaveragemarginaleffects(95% CI)	p–value
**Model 3 (Mobile phone access vs. no access)**								
*Among boys*								
No mobile phone access (Reference)	0	.	0	.	0	.	0	.
Mobile phone access	–0.0064 (−0.0453 to 0.0317)	0.731	–0.0021 (−0.0399 to 0.0352)	0.899	0.0546 (0.0125 to 0.0972)	0.011	–0.0066 (−0.0460 to 0.0328)	0.742
*Among girls*								
No mobile phone access (Reference)	0	.	0	.	0	.	0	.
Mobile phone access	–0.0062 (−0.0313 to 0.0187)	0.618	0.0125 (−0.0250 to 0.0487)	0.537	–0.0140 (−0.0451 to 0.0169)	0.359	0.0051 (−0.0257 to 0.0361)	0.659
*Among adolescents not living with HIV*								
No mobile phone access (Reference)	0	.	0	.	0	.	0	.
Mobile phone access	0.0004 (−0.0390 to 0.0399)	0.909	–0.0355 (−0.0853 to 0.0141)	0.151	0.0243 (−0.0212 to 0.0699)	0.296	0.0007 (−0.0401 to 0.0417)	0.919
*Among adolescents living with HIV*								
No mobile phone access (Reference)	0	.	0	.	0	.	0	.
Mobile phone access	–0.0105 (−0.0356 to 0.0150)	0.422	0.0293 (−0.0024 to 0.0609)	0.071	0.0063 (−0.0258 to 0.0380)	0.711	0.0017 (−0.0293 to 0.0321)	0.923
**Model 4 (Social media use and health content use vs. no mobile phone access)**								
*Among boys*								
No mobile phone access (Reference)	0	.	0	.	0	.	0	.
Social media alone	0.0655 (0.0113 to 0.1197)	0.015	0.0432 (−0.0052 to 0.0914)	0.077	0.0451 (−0.0045 to 0.0953)	0.073	0.0040 (−0.0447 to 0.0526)	0.873
Health content use	–0.0681 (−0.1145 to −0.0217)	0.004	−0.0684 (−0.1143 to −0.0225)	0.002	0.0775 (0.0160 to 0.1391)	0.014	0.0107 (−0.0491 to 0.0704)	0.723
*Among girls*								
No mobile phone access (Reference)	0	.	0	.	0	.	0	
Social media alone	0.0066 (−0.0203 to 0.0335)	0.630	0.0598 (0.0162 to 0.1034)	0.007	−0.0062 (−0.0389 to 0.0265)	0.710	0.0094 (−0.0241 to 0.0429)	0.582
Health content use	−0.0349 (−0.0631 to −0.0065)	0.016	−0.0803 (−0.1267 to −0.0340)	<0.001	−0.0273 (−0.0677 to 0.0131)	0.186	0.0242 (−0.0199 to 0.0683)	0.282
*Among adolescents not living with HIV*								
No mobile phone access (Reference)	0	.	0	.	0	.	0	
Social media alone	0.0046 (−0.0362 to 0.0455)	0.825	−0.0248 (−0.0771 to 0.0276)	0.354	0.0196 (−0.0269 to 0.0661)	0.409	−0.0133 (−0.0545 to 0.0280)	0.528
Health content use	−0.0196 (−0.0728 to 0.0335)	0.470	−0.1035 (−0.1731 to −0.0339)	0.004	0.0534 (−0.0266 to 0.1334)	0.191	0.0876 (0.0089 to 0.1663)	0.029
*Among adolescents living with HIV*								
No mobile phone access (Reference)	0	.	0	.	0	.	0	
Social media alone	0.0406 (0.0009 to 0.0700)	0.018	0.0943 (0.0533 to 0.1349)	0.001	0.0113 (−0.0258 to 0.0485)	0.551	0.0201 (−0.0167 to 0.0569)	0.284
Health content use	−0.0646 (−0.0876 to −0.0410)	0.001	–0.0601 (−0.0945 to –0.0257)	<0.001	–0.0092 (−0.0467 to 0.0284)	0.633	–0.0202 (−0.0550 to 0.0147)	0.256
N	1332		1351		1351		1333	
