# Supplementary material for: Associations of social media and health content use with sexual risk behaviours among adolescents in South Africa
Source: Sex Reprod Health Matters. 2023 Nov 10;31(4):2267893. doi: 10.1080/26410397.2023.2267893 (PMC10796125; doi:10.1080/26410397.2023.2267893)
Supplement: Supplemental Material: Appendices 1-6 [file ZRHM_A_2267893_SM6097.docx]

**Title: Associations of social media and health content use with sexual risk behaviours among adolescents in South Africa.**

**Appendix 1**

STROBE Statement—Checklist of items that should be included in reports of ***cohort studies***

|  | Item No | Recommendation | Page No |
| --- | --- | --- | --- |
| **Title and abstract** | 1 | (*a*) Indicate the study’s design with a commonly used term in the title or the abstract | 1 |
|  |  | (*b*) Provide in the abstract an informative and balanced summary of what was done and what was found | 2 |
| Introduction | | | |
| Background/rationale | 2 | Explain the scientific background and rationale for the investigation being reported | 3 |
| Objectives | 3 | State specific objectives, including any prespecified hypotheses | 3 |
| Methods | | | |
| Study design | 4 | Present key elements of study design early in the paper | 4 |
| Setting | 5 | Describe the setting, locations, and relevant dates, including periods of recruitment, exposure, follow-up, and data collection | 4/5 |
| Participants | 6 | (*a*) Give the eligibility criteria, and the sources and methods of selection of participants. Describe methods of follow-up | 4/5 |
|  |  | (*b*) For matched studies, give matching criteria and number of exposed and unexposed | N/A |
| Variables | 7 | Clearly define all outcomes, exposures, predictors, potential confounders, and effect modifiers. Give diagnostic criteria, if applicable | 4/6 |
| Data sources/ measurement | 8* | For each variable of interest, give sources of data and details of methods of assessment (measurement). Describe comparability of assessment methods if there is more than one group | 4/6 |
| Bias | 9 | Describe any efforts to address potential sources of bias | 6 |
| Study size | 10 | Explain how the study size was arrived at | 5 |
| Quantitative variables | 11 | Explain how quantitative variables were handled in the analyses. If applicable, describe which groupings were chosen and why | 6/7 |
| Statistical methods | 12 | (*a*) Describe all statistical methods, including those used to control for confounding | 6/7 |
|  |  | (*b*) Describe any methods used to examine subgroups and interactions | 6/7 |
|  |  | (*c*) Explain how missing data were addressed | 6 |
|  |  | (*d*) If applicable, explain how loss to follow-up was addressed | 6 |
|  |  | (*e*) Describe any sensitivity analyses | 6/7 |
| Results | | |  |
| Participants | 13* | (a) Report numbers of individuals at each stage of study—eg numbers potentially eligible, examined for eligibility, confirmed eligible, included in the study, completing follow-up, and analysed | 7/8 |
|  |  | (b) Give reasons for non-participation at each stage | 5/6 |
|  |  | (c) Consider use of a flow diagram | 5 |
| Descriptive data | 14* | (a) Give characteristics of study participants (eg demographic, clinical, social) and information on exposures and potential confounders | 7/8 |
|  |  | (b) Indicate number of participants with missing data for each variable of interest | N/A |
|  |  | (c) Summarise follow-up time (e.g., average and total amount) | N/A |
| Outcome data | 15* | Report numbers of outcome events or summary measures over time | 8/9 |
| Main results | 16 | (*a*) Give unadjusted estimates and, if applicable, confounder-adjusted estimates and their precision (eg, 95% confidence interval). Make clear which confounders were adjusted for and why they were included | 10/11 |
|  |  | (*b*) Report category boundaries when continuous variables were categorized | 10/11 |
|  |  | (*c*) If relevant, consider translating estimates of relative risk into absolute risk for a meaningful time period | N/A |
| Other analyses | 17 | Report other analyses done—e.g., analyses of subgroups and interactions, and sensitivity analyses | 10/13 |
| **Discussion** | | | |
| Key results | 18 | Summarise key results with reference to study objectives | 13/15 |
| Limitations | 19 | Discuss limitations of the study, taking into account sources of potential bias or imprecision. Discuss both direction and magnitude of any potential bias | 13/15 |
| Interpretation | 20 | Give a cautious overall interpretation of results considering objectives, limitations, multiplicity of analyses, results from similar studies, and other relevant evidence | 13/15 |
| Generalisability | 21 | Discuss the generalisability (external validity) of the study results | 14/15 |
| **Other information** | | | |
| Funding | 22 | Give the source of funding and the role of the funders for the present study and, if applicable, for the original study on which the present article is based | 19 |

*Give information separately for exposed and unexposed groups.

**Note:** An Explanation and Elaboration article discusses each checklist item and gives methodological background and published examples of transparent reporting. The STROBE checklist is best used in conjunction with this article (freely available on the Web sites of PLoS Medicine at http://www.plosmedicine.org/, Annals of Internal Medicine at http://www.annals.org/, and Epidemiology at http://www.epidem.com/). Information on the STROBE Initiative is available at http://www.strobe-statement.org.

**Appendix 2**

Comparison between participants interviewed in both waves (2 and 3) and those who dropped out of the study (between wave 2 and 3)

|  | Total (N=1,410) | Included (N=1,353) | Excluded (N=57) | p-value |
| --- | --- | --- | --- | --- |
| Rural residence | 27.4% | 27.5% | 26.3% | 0.85 |
| Informal housing | 18.0% | 17.8% | 22.8% | 0.34 |
| Poverty | 32.8% | 32.9% | 29.8% | 0.63 |
| Age (in years) | 13.8 (2.9) | 13.8 (3.0) | 14.5 (2.7) | 0.062 |
| Sex |  |  |  | 0.85 |
| Male | 43.3% | 43.4% | 42.1% |  |
| Female | 56.7% | 56.6% | 57.9% |  |
| ALHIV | 69.4% | 69.0% | 80.7% | 0.059 |
| In a relationship | 29.3% | 29.3% | 29.8% | 0.93 |
| School enrolment | 94.3% | 94.4% | 93.0% | 0.65 |
| Mobile phone use |  |  |  | 0.015 |
| No access | 49.6% | 49.8% | 43.9% |  |
| Social media only | 33.3% | 32.6% | 49.1% |  |
| Health content use | 17.1% | 17.6% | 7.0% |  |
| Sex after substance use | 7.2% | 7.1% | 9.8% | 0.46 |
| Multiple sexual partnerships | 15.1% | 15.2% | 12.3% | 0.54 |
| Unprotected sex | 14.7% | 14.5% | 19.3% | 0.31 |
| Inequitable sexual partnerships | 10.7% | 10.8% | 5.9% | 0.26 |

**Appendix 3**

**Missing data imputations**

**Table of missing data**

|  | Missing – N (%) |
| --- | --- |
| **Wave 1** |  |
| Female | 0 (0%) |
| ALHIV | 0 (0%) |
| Age | 0 (0%) |
| Rural residence | 0 (0%) |
| Informal housing | 1 (0.07%) |
| Poverty | 1 (0.07%) |
| In a relationship | 0 (0%) |
|  |  |
| **Wave 2, 2015–2017** |  |
| Mobile phone use | 0 (0%) |
| Sex after substance use | 183 (13.53%) |
| Unprotected sex | 0 (0%) |
| Multiple sexual partnerships | 0 (0%) |
| Inequitable sexual partnership (defined as self-report of sexual intercourse with partners 5+ years older than participants or in exchange of material gifts.) | 52 (3.84%) |
|  |  |
| **Wave 3, 2017–2018** |  |
| Mobile phone use | 0 (0%) |
| Sex after substance use | 88 (6.50%) |
| Unprotected sex | 0 (0%) |
| Multiple sexual partnerships | 0 (0%) |
| Inequitable sexual partnership | 116 (8.57%) |

**Determining whether the data on outcomes are MCAR**

Little's χ² test indicated that data were not missing completely at random Chi-square = 33.97 (with degrees of freedom 11) in wave 2 and 58.02 in wave 3 (with degrees of freedom 11)

**Appendix 4**

Spearman’s bivariate correlation coefficients (95% CI; p-value) between outcome variables. Below the diagonal (value 1) are between-participants correlations and above the diagonal are within-participants correlations

|  | Sex after substance use | Unprotected sex | Multiple sexual partnerships | Inequitable sexual partnerships |
| --- | --- | --- | --- | --- |
| Sex after substance use | 1 | 0.06 (0.02 to 0.10); p=0.003 | 0.16 (0.12 to 0.20); p<0.001 | 0.04 (0.00 to 0.08); p=0.043 |
| Unprotected sex | 0.25 (0.20 to 0.30); p<0.001 | 1 | 0.02 (-0.02 to 0.06); p=0.288 | 0.01 (-0.03 to 0.05); p=0.680 |
| Multiple sexual partnerships | 0.44 (0.40 to 0.48); p<0.001 | 0.38 (0.34 to 0.43); p<0.001 | 1 | 0.17 (0.13 to 0.21); p<0.001 |
| Inequitable sexual partnerships | 0.27 (0.22 to 0.32); p<0.001 | 0.39 (0.34 to 0.43); p<0.001 | 0.47 (0.42 to 0.51); p<0.001 | 1 |

**Appendix 5**

Summary of statistical models

| Model | Main association | Adjustment variables |
| --- | --- | --- |
| Model 1 | Average marginal effects from the multivariable mixed-effects logistic regression of individual sexual risk behaviours outcomes on mobile phone access (access vs. no access). | Participant’s sex, mean-centred age, HIV status, relationship status, and household poverty, rural residence, informal housing, school enrolment, and survey wave. |
| Model 2 | Average marginal effects from the multivariable mixed-effects logistic regression of individual sexual risk behaviours outcomes on mobile phone use (social media use alone and health content use vs. no access). | Participant’s sex, mean-centred age, HIV status, relationship status, and household poverty, rural residence, informal housing, school enrolment, and survey wave. |
| Model 3 | Average marginal effects from the multivariable mixed-effects logistic regression of individual sexual risk behaviours outcomes on mobile phone access (access vs. no access), participant’s sex, interaction between ownership, participant’s sex, and HIV status. Model 3 presents the average marginal effects from the association between mobile phone access and individual sexual risk behaviours outcomes for boys, girls, adolescents living with HIV, and not living with HIV. | Participant’s mean-centred age, relationship status, and household poverty, rural residence, informal housing, school enrolment, and survey wave. |
| Model 4 | Average marginal effects from the multivariable mixed-effects logistic regression of individual sexual risk behaviours outcomes on mobile phone use (social media use alone and health content use vs. no access), participant’s sex, interaction between ownership, participant’s sex, and HIV status. Model 4 presents the average marginal effects from the association between mobile phone use and individual sexual risk behaviours outcomes for boys, girls, adolescents living with HIV, and not living with HIV. | Participant’s mean-centred age, relationship status, and household poverty, rural residence, informal housing, school enrolment, and survey wave. |

**Appendix 6**

**Sensitivity analyses**

Multivariable association between mobile phone access and use and self-reported sexual risk behaviours. Results from mixed-effects logistic regression models on complete cases

|  | Sex after substance use | |  | Unprotected sex | |  | Multiple sexual partnerships | |  | Inequitable sexual partnerships | |
| --- | --- | --- | --- | --- | --- | --- | --- | --- | --- | --- | --- |
|  | Adjusted average marginal effects (95% CI) | p–value |  | Adjusted average marginal effects (95% CI) | p–value |  | Adjusted average marginal effects (95% CI) | p–value |  | Adjusted average marginal effects (95% CI) | p–value |
| **Model 1 (Mobile phone access vs. no access)** | | | | |  |  |  |  |  |  |  |
| No mobile phone access (Reference) | 0 | . |  | 0 | . |  | 0 | . |  | 0 | . |
| Mobile phone access | –0.0065 (–0.0271 to 0.0140) | 0.551 |  | 0.0062 (–0.0201 to 0.0335) | 0.611 |  | 0.0153 (–0.0109 to 0.0411) | 0.242 |  | 0.0005 (–0.0247 to 0.0250) | 0.961 |
| **Model 2 (Social media alone and health content vs. no mobile phone access)** |  |  |  |  |  |  |  |  |  |  |  |
| No mobile phone access (Reference) | 0 | . |  | 0 | . |  | 0 | . |  | 0 | . |
| Social media alone | 0.0252 (–0.0021 to 0.0482) | 0.077 |  | 0.0456 (0.0142 to 0.0767) | 0.005 |  | 0.0184 (–0.0105 to 0.0465) | 0.209 |  | 0.0017 (–0.0251 to 0.0285) | 0.905 |
| Health content use | –0.0538 (–0.0746 to –0.0331) | <0.001 |  | –0.0758 (–0.1072 to –0.0445) | <0.001 |  | 0.0107 (–0.0220 to 0.0433) | 0.534 |  | –0.0016 (–0.0322 to 0.0291) | 0.919 |
| N | 1332 |  |  | 1351 |  |  | 1351 |  |  | 1333 |  |

Comparisons of the multivariable association between mobile phone access and use and self-reported sexual risk behaviours for boys, girls, adolescents living with and without HIV. Results from mixed-effects logistic regression models on complete cases

|  | Sex after substance use | |  | Unprotected sex | |  | Multiple sexual partnerships | |  | Inequitable sexual partnerships | |
| --- | --- | --- | --- | --- | --- | --- | --- | --- | --- | --- | --- |
|  | Adjusted average marginal effects (95% CI) | p–value |  | Adjusted average marginal effects (95% CI) | p–value |  | Adjusted average marginal effects (95% CI) | p–value |  | Adjusted average marginal effects (95% CI) | p–value |
| **Model 3 (Mobile phone access vs. no access)** | | | | |  |  |  |  |  |  |  |
| *Among boys* | |  |  |  |  |  |  |  |  |  |  |
| No mobile phone access (Reference) | 0 | . |  | 0 | . |  | 0 | . |  | 0 | . |
| Mobile phone access | –0.0064 (–0.0453 to 0.0317) | 0.731 |  | –0.0021 (–0.0399 to 0.0352) | 0.899 |  | 0.0546 (0.0125 to 0.0972) | 0.011 |  | –0.0066 (–0.0460 to 0.0328) | 0.742 |
| *Among girls* |  |  |  |  |  |  |  |  |  |  |  |
| No mobile phone access (Reference) | 0 | . |  | 0 | . |  | 0 | . |  | 0 | . |
| Mobile phone access | –0.0062 (–0.0313 to 0.0187) | 0.618 |  | 0.0125 (–0.0250 to 0.0487) | 0.537 |  | –0.0140 (–0.0451 to 0.0169) | 0.359 |  | 0.0051 (–0.0257 to 0.0361) | 0.659 |
| *Among adolescents not living with HIV* | | | | |  |  |  |  |  |  |  |
| No mobile phone access (Reference) | 0 | . |  | 0 | . |  | 0 | . |  | 0 | . |
| Mobile phone access | 0.0004 (–0.0390 to 0.0399) | 0.909 |  | –0.0355 (–0.0853 to 0.0141) | 0.151 |  | 0.0243 (–0.0212 to 0.0699) | 0.296 |  | 0.0007 (–0.0401 to 0.0417) | 0.919 |
| *Among adolescents living with HIV* | | | | |  |  |  |  |  |  |  |
| No mobile phone access (Reference) | 0 | . |  | 0 | . |  | 0 | . |  | 0 | . |
| Mobile phone access | –0.0105 (–0.0356 to 0.0150) | 0.422 |  | 0.0293 (–0.0024 to 0.0609) | 0.071 |  | 0.0063 (–0.0258 to 0.0380) | 0.711 |  | 0.0017 (–0.0293 to 0.0321) | 0.923 |
| **Model 4 (Social media use and health content use vs. no mobile phone access)** | | | | | | | | | | |  |
| *Among boys* | |  |  |  |  |  |  |  |  |  |  |
| No mobile phone access (Reference) | 0 | . |  | 0 | . |  | 0 | . |  | 0 | . |
| Social media alone | 0.0655 (0.0113 to 0.1197) | 0.015 |  | 0.0432 (–0.0052 to 0.0914) | 0.077 |  | 0.0451 (–0.0045 to 0.0953) | 0.073 |  | 0.0040 (–0.0447 to 0.0526) | 0.873 |
| Health content use | –0.0681 (–0.1145 to –0.0217) | 0.004 |  | –0.0684 (–0.1143 to –0.0225) | 0.002 |  | 0.0775 (0.0160 to 0.1391) | 0.014 |  | 0.0107 (–0.0491 to 0.0704) | 0.723 |
| *Among girls* |  |  |  |  |  |  |  |  |  |  |  |
| No mobile phone access (Reference) | 0 | . |  | 0 | . |  | 0 | . |  | 0 |  |
| Social media alone | 0.0066 (–0.0203 to 0.0335) | 0.630 |  | 0.0598 (0.0162 to 0.1034) | 0.007 |  | –0.0062 (–0.0389 to 0.0265) | 0.710 |  | 0.0094 (–0.0241 to 0.0429) | 0.582 |
| Health content use | –0.0349 (–0.0631 to –0.0065) | 0.016 |  | –0.0803 (–0.1267 to –0.0340) | <0.001 |  | –0.0273 (–0.0677 to 0.0131) | 0.186 |  | 0.0242 (–0.0199 to 0.0683) | 0.282 |
| *Among adolescents not living with HIV* |  |  |  |  |  |  |  |  |  |  |  |
| No mobile phone access (Reference) | 0 | . |  | 0 | . |  | 0 | . |  | 0 |  |
| Social media alone | 0.0046 (–0.0362 to 0.0455) | 0.825 |  | –0.0248 (–0.0771 to 0.0276) | 0.354 |  | 0.0196 (–0.0269 to 0.0661) | 0.409 |  | –0.0133 (–0.0545 to 0.0280) | 0.528 |
| Health content use | –0.0196 (–0.0728 to 0.0335) | 0.470 |  | –0.1035 (–0.1731 to –0.0339) | 0.004 |  | 0.0534 (–0.0266 to 0.1334) | 0.191 |  | 0.0876 (0.0089 to 0.1663) | 0.029 |
| *Among adolescents living with HIV* |  |  |  |  |  |  |  |  |  |  |  |
| No mobile phone access (Reference) | 0 | . |  | 0 | . |  | 0 | . |  | 0 |  |
| Social media alone | 0.0406 (0.0009 to 0.0700) | 0.018 |  | 0.0943 (0.0533 to 0.1349) | 0.001 |  | 0.0113 (–0.0258 to 0.0485) | 0.551 |  | 0.0201 (–0.0167 to 0.0569) | 0.284 |
| Health content use | –0.0646 (–0.0876 to –0.0410) | 0.001 |  | –0.0601 (–0.0945 to –0.0257) | <0.001 |  | –0.0092 (–0.0467 to 0.0284) | 0.633 |  | –0.0202 (–0.0550 to 0.0147) | 0.256 |
| N | 1332 |  |  | 1351 |  |  | 1351 |  |  | 1333 |  |
